# Role of Autophagy in Goose Astrovirus-Induced Renal Injury in Goslings

**DOI:** 10.3390/ani16142214

**Published:** 2026-07-16

**Authors:** Jun Kuang, Zhenni Liu, Haoyu Huang, Yan Shi, Meiqin Wu, Zhixian Wang, Xiaona Gao, Xiaoquan Guo, Xinjun Liao, Haiqin Li

**Affiliations:** 1 School of Life Sciences, Key Laboratory of Jiangxi Province for Biological Invasion and Biosecurity, Key Laboratory of Jiangxi Province for Functional Biology and Pollution Control in Red Soil Regions, Jinggangshan University, Ji’an 343009, China; kuangjun1986919@163.com; 2 Jiangxi Provincial Key Lab for Animal Health, College of Animal Science and Technology, Jiangxi Agricultural University, Nanchang 330045, China; vetzhenny@163.com (Z.L.); icehaoyu@foxmail.com (H.H.); shiyan0015@126.com (Y.S.); 18379391191@163.com (M.W.); wangzx0530@126.com (Z.W.); xiaona.gao@jxau.edu.cn (X.G.); xqguo20720@jxau.edu.cn (X.G.); 3 Institute of Animal Husbandry and Veterinary Medicine, Jiangxi Academy of Agricultural Sciences, Nanchang 330200, China

**Keywords:** goose astrovirus, renal injury, autophagy

## Abstract

Goose astrovirus (GoAstV) is a newly identified virus affecting geese, leading to goose gout, which is marked by urate deposits in organs and joints. This research investigates the role of autophagy in renal damage caused by GoAstV in goslings. The findings indicated that GoAstV infection in goslings resulted in characteristic clinical manifestations, with renal tissues displaying tubular swelling, inflammatory infiltration, and autophagosome formation. In vivo experiments demonstrated a significant upregulation of mRNA levels for autophagy-related factors, including *AMPK*, *LC3A*, *ATG5*, *ATG7*, *P62*, *Beclin1*, *AMBRA1* and *GABARAPL1*, while *mTOR* and *LC3B* levels were notably decreased. At 3 dpi, the protein expression levels of ATG5, Beclin1, and LC3B II/I increased, while P62 levels decreased, suggesting autophagy activation. In vitro analyses revealed that GoAstV infection led to enhanced autophagy; however, the concurrent upregulation of LC3B II/I and P62 proteins suggested an obstruction in the autophagic flux. Upon the inhibition of autophagy with 3-methyladenine (3-MA, autophagy inhibitor), there was a significant reduction in the expression of autophagy-related factors, accompanied by a marked decrease in viral replication rates. In conclusion, GoAstV infection in gosling kidney cells initially triggers enhanced autophagy and subsequently causes a blockage in the autophagic flux. The virus exploits autophagosomes for replication, ultimately resulting in kidney damage. The application of 3-MA effectively inhibits this autophagic process and diminishes viral replication.

## Simple Summary

This research offers new insights into the pathogenesis of GoAstV infection in gosling kidneys, revealing that the infection initially triggers autophagy but later obstructs autophagic flux, as evidenced by the elevated levels of LC3B and P62 levels in vitro. The virus utilizes autophagosomes to facilitate its replication, leading to renal damage. The application of 3-MA, an autophagy inhibitor, effectively reduces viral replication, suggesting a potential antiviral strategy against GoAstV-induced gout.

## 1. Introduction

Astroviruses (AstVs) are non-enveloped, single-stranded positive-sense RNA viruses from the Astroviridae family, infecting various mammalian and avian hosts [1]. This family is divided into two genera: Mamastrovirus, mainly affecting mammals including humans, and Avastrovirus, primarily infecting avian species like chickens, ducks, and geese [2]. Astroviruses frequently cause acute gastroenteritis in humans, primarily impacting young children and the elderly, with rare cases of encephalitis [3]. In avian species, astrovirus infections are linked to a variety of pathological conditions, including enteritis mortality syndrome, growth retardation, broiler dwarfism, white-feathered syndrome, visceral gout, and fatal hepatitis in ducklings [4]. A newly discovered goose astrovirus (GoAstV) causes gout in goslings, characterized by joint swelling, urate buildup in organs and joints, and a 50% mortality rate [5]. The primary target organs of GoAstV include the liver, spleen, kidneys, and intestines, with the kidneys being the principal target [6]. Affected goslings exhibit severe visceral gout, with abundant uric acid crystals enveloping the heart and liver, as well as enlarged, urate-laden kidneys [7].

Autophagy is a conserved catabolic process crucial for cellular homeostasis, recycling intracellular components through a lysosome-dependent mechanism [8]. This process is pivotal in regulating energy metabolism and ensuring cellular quality control [9]. The core machinery of autophagy is modulated by upstream signaling pathways, particularly the AMPK-mTOR axis, which monitors cellular energy status [10]. Downstream effectors, such as the class III PI3K complex (comprising Beclin1 and AMBRA1), the ATG5-ATG12-ATG16L1 ubiquitin-like conjugation system, and the lipidation of LC3/GABARAP family proteins, coordinate the nucleation, elongation, and closure of autophagosomes [11]. The selective autophagy receptor p62 recognizes ubiquitinated cargo and links it to LC3/GABARAP proteins, promoting targeted degradation [12]. Autophagy plays a dual role in viral infections, either inhibiting viral replication by degrading viral components or being exploited by viruses to enhance their propagation [13]. Considering that the kidney serves as the primary target organ for GoAstV and that renal damage is pivotal in the pathogenesis of gout, it is crucial to comprehend the cellular mechanisms underlying GoAstV-induced renal injury [14]. Dysregulated autophagy has been implicated in various renal diseases and disorders of urate metabolism [15]. Research has shown that the foot-and-mouth disease virus (FMDV) facilitates the degradation of host proteins via macroautophagy/autophagy, consequently enhancing viral replication [16]. Similarly, infection with the porcine reproductive and respiratory syndrome virus (PRRSV) triggers macroautophagy/autophagy, thereby promoting viral replication [13]. Additionally, goose nephritic astrovirus infection elevates autophagy levels, disrupts intercellular junctions in renal tubular epithelial cells, and causes damage to podocytes in the kidneys of infected goslings [17]. However, the exact function of autophagy in the development of GoAstV, especially in relation to renal tubular damage and gout, is not well comprehended.

Since 2016, GoAstV has led to high-mortality outbreaks in Chinese goose-rearing regions, causing significant economic losses [18]. Although existing research has identified the kidney as a major target organ and linked renal impairment to exacerbated gout, the extent to which autophagy contributes to GoAstV-induced renal damage has not been systematically explored. Understanding the role of autophagy in astrovirus replication is crucial for developing effective prevention and control strategies. This study investigated how cellular autophagy affects gosling kidney damage caused by GoAstV. Animal experiments simulated natural infections, and tissue samples were collected over time. Q-PCR was used to assess autophagy and viral load in goose kidneys. An in vitro model with primary goose renal cells was also developed to study the virus-induced autophagic response, using the inhibitor 3-methyladenine (3-MA, autophagy inhibitor) to test whether blocking autophagy reduces GoAstV effects. This research sheds light on autophagy’s role in GoAstV-related kidney damage and lays the groundwork for future anti-GoAstV treatments.

## 2. Materials and Methods

### 2.1. Animal Feeding and Sample Collection

In this study, 100 healthy 1-day-old Xingguo gray goslings (Xingguo Grey Goose Breeding Farm, Ganzhou, China) were randomly assigned to either a control group or a GoAstV-infected group, with each group housed in separate, isolated rooms. Environmental conditions, including temperature and humidity, were consistently maintained across both rooms, and an identical photoperiod was regulated. On the inoculation day, 100 goslings in the GoAstV group were subcutaneously injected with 0.2 mL of GoAstV isolated and characterized by Jiangxi Academy of Agricultural Sciences (GoAstV-JX01 strain, NCBI number: MZ576222.1) at 2 × 10^6^ TCID_50_, while the control group received 0.2 mL of saline. Kidney samples were obtained from six goslings per group at 1, 3, 6, and 9 days post-infection (dpi). Throughout the experimental period, both groups had unrestricted access to water and feed, with the basal diet formulated in strict accordance with NRC standards.

### 2.2. Cell Culture

Xingguo gray goslings, aged between 7 and 14 days, were selected for this study. The subjects were decapitated and subsequently immersed in 75% ethanol for a duration of 5 min prior to being transferred to a laminar flow hood. Under sterile conditions, the sacrum and both kidneys were excised, followed by the removal of blood vessels, ureters, and connective tissue. The kidney tissue was then finely minced and washed three times with PBS. The digestion process involved incubating a mixture with 1 mg/mL type I collagenase in a 37 °C water bath for 8 min. Digestion was halted by adding an equal volume of DMEM with 10% fetal bovine serum. The resultant mixture was gently pipetted and filtered through a 200-mesh filter. The filtrate underwent centrifugation at 1200 rpm for 10 min, followed by the removal of the supernatant. The cell pellet was resuspended in erythrocyte lysis buffer, followed by a 10 min centrifugation, and the supernatant was discarded. A wash with DMEM was performed. The cell pellet was resuspended in DMEM supplemented with 10% fetal bovine serum (FBS) and 1% penicillin–streptomycin. FBS concentration was reduced to 2% during infection. Cells were seeded into culture plates at a density of 1 × 10^5^ cells/mL. The cultures were incubated in a 37 °C incubator with 5% CO_2_.

### 2.3. Hematoxylin–Eosin (H&E) Staining

The fixed kidney tissue was processed by trimming it to create a flat surface, followed by dehydration using a dehydrator. Subsequently, the tissue was infiltrated and embedded in paraffin. The paraffin block was then trimmed, and sections were cut at a thickness of 4 μm. These sections were spread and baked for subsequent use. Post dewaxing and rehydration, the sections were fixed in a tissue fixative for 15 min and subjected to a high-definition pre-staining solution for 1 min. Staining was performed with hematoxylin for 5 min, followed by differentiation using a differentiation solution and re-staining with a re-staining solution. The sections underwent dehydration through an ethanol gradient and were stained with eosin for 15 s. The sections were dehydrated for transparency and mounted with neutral resin.

### 2.4. Transmission Electron Microscopy

A 1 mm^3^ kidney tissue sample was washed three times with phosphate-buffered saline (PBS), each for 15 min. The tissue was fixed for 2 h at room temperature in darkness using a PBS solution with 1% osmium tetroxide. After fixation, three additional washes were performed with PBS. The tissue was dehydrated through a graded ethanol series, with each step lasting 20 min, followed by two 15 min immersions in acetone. The tissue was embedded in epoxy resin. Ultrathin sections with a thickness of 60–80 nm were prepared. The sections were stained with a 2% uranyl acetate solution and a 2.6% lead citrate solution. The sections were left to dry at room temperature overnight prior to examination with a transmission electron microscope.

### 2.5. Immunofluorescent Co-Localization of LC3B and Cap

Cells were cultured on slides until they achieved around 50% confluence before proceeding with viral infection. The slides were rinsed with PBS, PBS solution containing 0.1% Triton X-100 was applied, and incubation was conducted at room temperature for 20 min; then the slides were washed thrice with PBS. A mixture of LC3B and Cap primary antibodies was introduced, and incubation was conducted overnight (≥12 h) in a shaking incubator at 4 °C. Following incubation, the slides were rinsed thrice with PBS, and a suitable fluorescent secondary antibody was applied (goat anti-mouse/anti-rabbit IgG mixture), incubating at room temperature for 50 min. Three PBS washes were conducted, DAPI was applied for nuclear staining, and incubation was conducted in the dark at room temperature for 10 min. Three PBS washes were performed, slides were mounted with anti-fade medium, and analysis was conducted using a fluorescence microscope.

### 2.6. Detection of Cellular Autophagy

Cells were placed in culture plates and infected with the virus once they reached approximately 50% confluence. Following treatment, the cells were collected as outlined in Section 2.2. The CYTO-ID^®^ Autophagy Detection Kit 2.0 was applied (Enzo Life Sciences, New York, NY, USA), detecting autophagy in cells. Treatment was conducted with the staining solution of the kit, and the cells were incubated at room temperature in the dark for 30 min. The cells were centrifuged at 500× *g* for 5 min, the supernatant was discarded, the cells were washed once with staining buffer, and they were then resuspended. Autophagy levels were immediately analyzed using a flow cytometer.

### 2.7. Extraction of Total RNA and Quantitative Real-Time PCR Analysis

Cryopreserved gosling kidney tissue or cells were mixed with 1 mL of TRIzol to extract total RNA, whose purity and concentration were measured using a microspectrophotometer. RNA was converted to cDNA using a reverse transcription kit, combined with Mix, and stored at −80 °C. The cDNA was then linked with primers, and target gene expression was calculated using the 2^−ΔΔCT^ formula. Primers were synthesized by Beijing Tsingke Biotech Co., Ltd. (Beijing, China), using sequences from GenBank via the NCBI website, as detailed in Table 1.

### 2.8. Western Blotting

Protein extraction from gosling kidneys or cells was performed using RIPA lysis buffer (Solarbio Science & Technology Co., Ltd., Beijing, China). Following cell lysis, protein quantification was performed using the BCA method. The primary antibodies of ATG5, LC3II/LC3I, Beclin1, P62 and GAPDH were obtained from Wanlei Biotechnology Co. (Shenyang, China). The primary antibodies were diluted as follows: GAPDH, 1:5000; ATG5, 1:1000; LC3II/LC3I, 1:1000; Beclin1, 1:1000; and P62, 1:1000. Proteins were gel-separated by molecular weight, transferred to a PVDF membrane through wet transfer, and visualized with a Bio-Rad ChemiDoc chemiluminescence system (Des Plaines, IL, USA). ImageJ software 1.53 was used to analyze the target bands.

### 2.9. Statistical Analysis

Following the organization and summarization of the data using Excel 2021, statistical analyses were conducted utilizing GraphPad Prism 9.3. The analyses utilized independent samples *t*-tests and a one-way ANOVA, depending on sample selection. The data and their differences were graphically represented, with the results shown as the mean ± standard deviation (mean ± SD). In this study, an asterisk (*) indicates a statistically significant difference (*p* < 0.05), and a double asterisk (**) represents a highly significant difference (*p* < 0.01).

## 3. Results

### 3.1. Kidney Injury Induced by Goose Astrovirus

**Figure 1:**
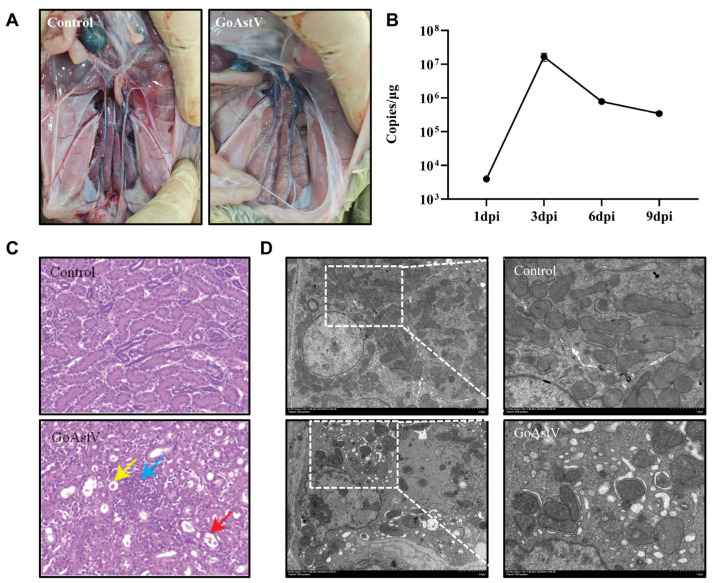
Effects of GoAstV in kidney injury in gosling. (**A**): Anatomy of gosling infected with GoAstV. (**B**): Changes in viral load of GoAstV during kidney infection. (**C**): HE staining of kidney of gosling infected with GoAstV. Note: Blue arrows indicate inflammatory infiltration; yellow arrows denote luminal exudate; red arrows represent glomerular damage. (**D**): Changes in viral load of GoAstV during kidney infection.

Figure 1A shows that three days post-infection with GoAstV, infected goslings displayed lethargy, loss of appetite, a tendency to lie down, and white, watery feces compared to the control group. Necropsy revealed enlarged livers and spleens, with significant renal damage: pale, enlarged kidneys with urate deposits and a brittle texture. Figure 1B indicates that the viral load in kidneys peaked at 10^7^ copies/μg at 3 dpi, then gradually declined, confirming GoAstV replication in the kidneys. Histopathological changes in kidney tissue were examined using HE staining at the viral load peak. In the control group (Figure 1C), renal cells were compact with clear borders and showed no signs of pathology. Conversely, the infected group exhibited significant tubular swelling, exudate in the tubular lumen, inflammatory cell clusters, and glomerular fragmentation, indicating kidney damage in goslings post-GoAstV infection. Transmission electron microscopy at 3 dpi, the peak of viral load, revealed further ultrastructural kidney changes (Figure 1D). In the control group, cell nuclei were oval with intact membranes and uniform chromatin, while mitochondria were densely packed with regular structures. Few small autophagosomes were present. In contrast, infected cells showed shrunken, deformed nuclei with aggregated chromatin; severely damaged mitochondria with swollen and fragmented cristae; and numerous double-membrane structures and mature autophagosomes.

### 3.2. Autophagy in Goose Astrovirus-Induced Kidney Injury in Goslings

**Figure 2:**
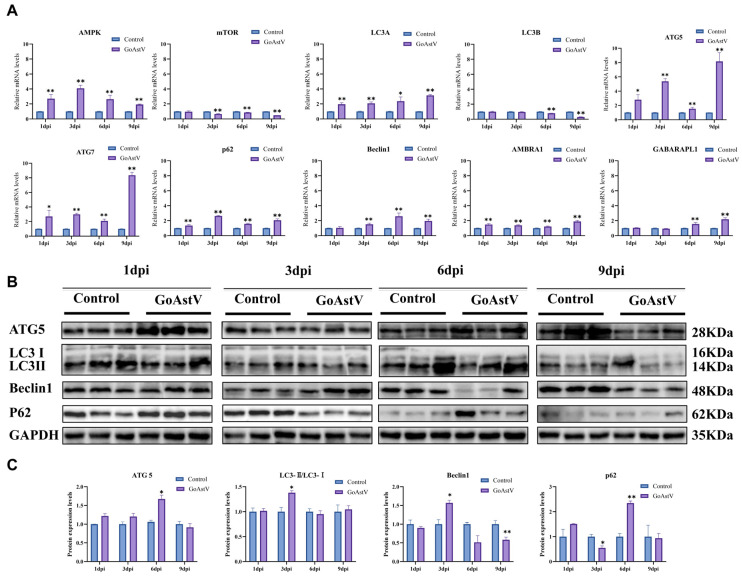
Effects of GoAstV on autophagy levels in kidney of gosling. (**A**): q-PCR results of autophagy-related mRNA. (**B**): Western blot result of autophagy-related protein levels in kidney of gosling. (**C**): Relative expression level of autophagy-related protein in kidney of gosling. *p* < 0.05 (*), *p* < 0.01 (**).

Figure 2A illustrates a significant increase in the mRNA levels of *AMPK*, *LC3A*, *ATG5*, *ATG7*, *P62*, and *AMBRA1* at 1, 3, 6, and 9 dpi, while *mTOR* mRNA levels significantly decreased at 3, 6, and 9 dpi, and *LC3B* mRNA levels decreased at 6 and 9 dpi (*p* < 0.05 or *p* < 0.01). *GABARAPL1* mRNA levels increased at 6 and 9 dpi (*p* < 0.05 or *p* < 0.01). Figure 2B shows the Western blot results. Figure 2C reveals that ATG5 protein levels significantly rose at 6 dpi, LC3II/I protein levels increased at 3 dpi, Beclin1 protein levels increased at 3 dpi and decreased at 9 dpi, and P62 protein levels decreased at 3 dpi and increased at 6 dpi (*p* < 0.05 or *p* < 0.01).

### 3.3. Effects of GoAstV in Primary Renal Tubular Epithelial Cells of Gosling

**Figure 3:**
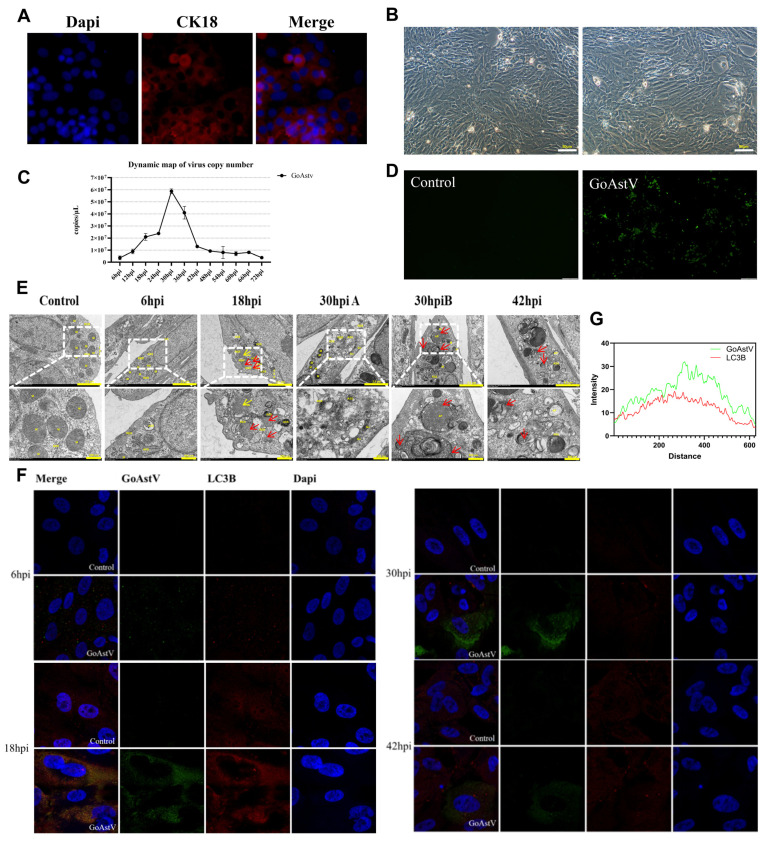
Effects of GoAstV in primary renal tubular epithelial cell of gosling. (**A**): Representative immunofluorescence image of CK18 staining (200×). Red presents cytokeratin18 (CK18). (**B**): Infection of primary renal tubular epithelial cells with GoAstV (200×). (**C**): Dynamic changes in viral load of GoAstV-infected primary renal tubular epithelial cells. (**D**): Immunofluorescence staining results of GoAstV (100×). Green represents Cap of GoAstV. (**E**): Effects of GoAstV infection on ultrastructure of primary renal tubular epithelial cells in geese. N represents nucleus; M represents mitochondria; RER represents rough endoplasmic reticulum; ASS represents autophagolysosome; AP represents autophagosome; red arrows indicate viral particles; yellow arrows indicate release of viral particles. (**F**): Effects of GoAstV infection on LC3B and Cap protein co-localization (600×). DAPI staining shows blue fluorescence for cell nuclei, while red and green fluorescence indicate LC3B and GoAstV Cap protein, respectively. (**G**): Quantitative statistical analysis of co-localization of LC3B and GoAstV Cap protein at 30 hpi.

Figure 3A demonstrates that the isolated primary cells attained a high level of purity, with CK18 positivity exceeding 80%. As shown in Figure 3B, renal tubular epithelial cells infected with GoAstV at an MOI of 1 showed shrinkage, aggregation, vacuolization, and significant detachment after 24 h, unlike the control group. This indicates a successful infection model. GoAstV replication in primary goose renal cells was monitored using absolute quantitative PCR, revealing rapid viral replication between 12 and 30 h, peaking at 30 h, and declining after 36 h, as shown in Figure 3C. Wells showing green fluorescence were marked as positive (Figure 3D). As shown in Figure 3E, cells had clear borders, intact organelles, and no autophagy-related structures. At 6 hpi, cells showed slight organelle swelling and autophagosome-like vesicles. Astroviruses are single-stranded positive-stranded RNA viruses, and viral particles are visible under electron microscopy as a star-shaped structure with a diameter of 20–30 nm. By 18 hpi, cells became irregular with numerous autophagosomes and viral particles in double-membrane vesicles (DMVs, red arrow). At 30 hpi in Field A, cells had blurred membranes and damaged mitochondria, while in Field B, nuclei were irregular, and organelles were swollen with vacuoles. By 42 hpi, cell membranes remained intact, but organelles were swollen, with many vacuoles and fragmented mitochondrial cristae. Numerous autophagosomes and some autolysosomes contained a few viral particles. Figure 3F,G show that in GoAstV-infected renal tubular epithelial cells, GoAstV fluorescence rises at 6, 18, and 30 hpi, then declines at 42 hpi. At 18 hpi, LC3B and GoAstV co-localize (yellow), indicating a link between viral replication and autophagy.

### 3.4. Autophagy in Primary Renal Tubular Epithelial Cells of Gosling Induced by GoAstV

**Figure 4:**
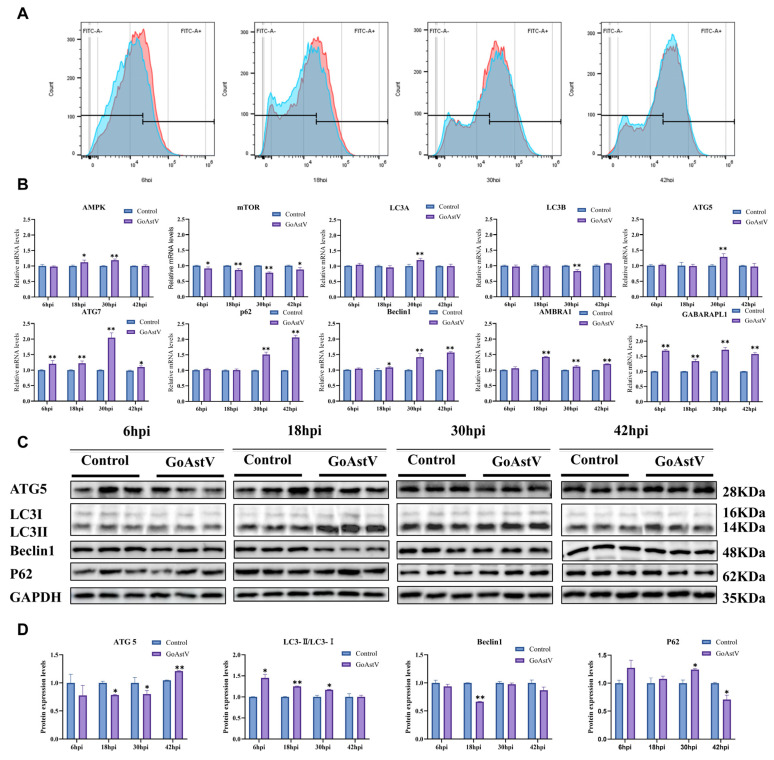
Effects of GoAstV on autophagy levels in renal primary renal tubular epithelial cells of gosling. (**A**): Effects of GoAstV infection on cellular autophagy. Blue represents the control group, red represents the GoAstV group. (**B**): Effects of GoAstV infection on autophagy-related mRNA expression in renal tubular epithelial cells. (**C**): Western blot result of autophagy-related protein levels in renal primary renal tubular epithelial cells. (**D**): Relative expression level of autophagy-related protein in renal primary renal tubular epithelial cells. *p* < 0.05 (*), *p* < 0.01 (**).

As shown in Figure 4A, compared to the control group, the fluorescence distribution patterns at 6, 18, and 30 hpi shifted to the right following GoAstV infection, with no significant difference observed at 42 hpi. This indicates that GoAstV infection induces enhanced autophagy during the early stages of infection. As illustrated in Figure 4B, the mRNA expression levels of *AMPK* were significantly elevated at 30 and 42 hpi (*p* < 0.05 or *p* < 0.01). Conversely, the mRNA levels of *mTOR* were significantly reduced at 6, 18, 30, and 42 hpi (*p* < 0.05 or *p* < 0.01). The expression levels of *LC3A* and *ATG5* mRNA were notably increased at 30 hpi (*p* < 0.01), while *LC3B* mRNA levels were significantly decreased at the same time point (*p* < 0.01). Additionally, the mRNA levels of *ATG7* and *GABARAPL1* exhibited significant increases at 6, 18, 30, and 42 hpi (*p* < 0.05 or *p* < 0.01). The mRNA expression of *P62* was significantly elevated at 30 and 42 hpi (*p* < 0.01). Furthermore, the mRNA levels of *Beclin1* and *AMBRA1* were significantly increased at 18, 30, and 42 hpi (*p* < 0.05 or *p* < 0.01). Figure 4B presents the results of the Western blotting assay. As illustrated in Figure 4C, the relative protein levels of ATG5 were significantly reduced at 18, 30, and 42 hpi (*p* < 0.05 or *p* < 0.01). Conversely, the relative protein levels of LC3II/LC3I were significantly elevated at 6, 18, and 30 hpi (*p* < 0.05 or *p* < 0.01). The relative protein level of Beclin1 showed a significant decrease at 18 hpi (*p* < 0.01), whereas the relative protein level of P62 demonstrated a significant increase at 30 hpi (*p* < 0.05) and a significant decrease at 42 hpi (*p* < 0.05).

### 3.5. Effects of 3-MA on GoAstV-Induced Autophagy

**Figure 5:**
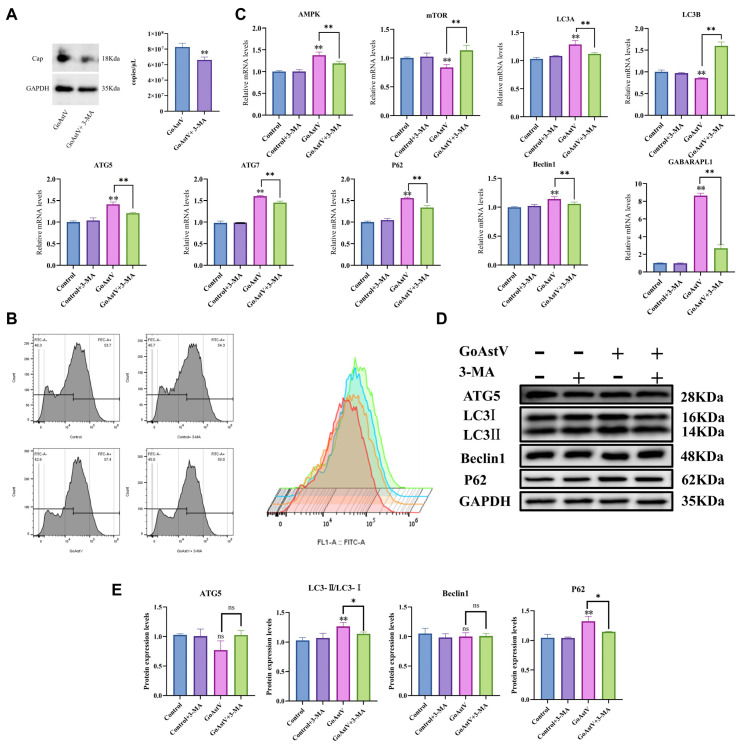
Effects of 3-MA on GoAstV-induced autophagy. (**A**): Effect of 3-MA on GoAstV replication. (**B**): Effect of 3-MA on GoAstV infection-induced cellular autophagy. Color code: red (Control group), orange (Control + 3-MA group), blue (GoAstV group), green (GoAstV + 3-MA group). (**C**): Effect of PI3K inhibition on autophagy-related mRNA expression in GoAstV-infected renal tubular epithelial cells. (**D**): WB results of PI3K inhibition on autophagy-related protein. (**E**): WB analysis of PI3K inhibition on autophagy-related protein. *p* > 0.05 (ns, not significant), *p* < 0.05 (*), *p* < 0.01 (**).

Primary goose renal tubular epithelial cells infected with GoAstV were subjected to treatment with the autophagy inhibitor 3-methyladenine (3-MA) for 30 hpi, followed by the collection of cell samples. As illustrated in Figure 5A, the expression levels of the GoAstV Cap protein and its corresponding gene were significantly diminished in the GoAstV-infected cells treated with 3-MA compared to the untreated GoAstV-infected group (*p* < 0.01). Figure 5B illustrates that, relative to the control group, the GoAstV group images shifted right with more positive cells. In contrast, the GoAstV+3-MA group images shifted left, showing a reduced positive rate. This suggests that the autophagy inhibitor 3-MA can reduce cellular autophagy induced by GoAstV infection. As shown in Figure 5C, upon infection with GoAstV, there was a significant upregulation in the mRNA expression levels of *AMPK*, *LC3A*, *ATG5*, *ATG7*, *P62*, *Beclin1*, and *GABARAPL1* (*p* < 0.01), while the mRNA levels of *mTOR* and *LC3B* were significantly downregulated (*p* < 0.01). However, treatment with 3-MA effectively reversed these changes (*p* < 0.01). The results of the Western blotting assay are depicted in Figure 5D. As shown in Figure 5E, GoAstV infection led to a marked increase in the relative protein levels of LC3II/LC3I and P62 (*p* < 0.01), which was subsequently reversed by 3-MA treatment (*p* < 0.05).

## 4. Discussion

The novel goose astrovirus (GoAstV) induces clinical manifestations in goslings, including pyrexia, articular swelling, lethargy, and anorexia. Postmortem analyses reveal urate accumulation in the hepatic and renal tissues, as well as within the abdominal cavity, accompanied by renal hypertrophy [19]. The mortality rate among affected goslings reached up to 50%, leading to substantial economic losses. Currently, there are no specific antiviral treatments or approved vaccines available for GoAstV infection, with control strategies predominantly relying on biosecurity measures [20]. Prior research has demonstrated that the GoAstV-II strain possesses greater infectivity and lethality compared to the GoAstV-I strain, predominantly targeting the kidneys, with renal tubular epithelial cells being the principal cell type affected [21]. Nevertheless, the majority of previous studies have predominantly utilized animal models or passaged cell lines, which do not adequately replicate the in vivo microenvironment of primary goose renal tubular epithelial cells. This limitation has significantly impeded a thorough understanding of the mechanisms underlying viral pathogenesis. In this study, primary goose renal tubular epithelial cells were isolated from goslings aged 7 to 14 days, achieving high purity (CK18 positivity > 80%) and viability. Furthermore, an infection system was developed for the GoAstV-JX01 (GoAstV-II) strain using this cellular model. Utilizing this in vitro model to accurately assess viral replication dynamics, the results revealed that upon the infection of primary goose renal tubular epithelial cells with GoAstV-JX01 at a multiplicity of infection (MOI) of 1, the viral load reached its maximum at 30 hpi before undergoing a rapid decline.

Autophagy, an evolutionarily conserved lysosome-mediated degradation pathway, is crucial for maintaining eukaryotic cell homeostasis during stress conditions like nutrient deprivation, oxidative stress, or pathogen invasion [22]. In this process, cells generate double-membrane autophagosomes to sequester damaged organelles, misfolded proteins, or invading pathogens [8]. These autophagosomes subsequently fuse with lysosomes to form autolysosomes, where the sequestered contents are degraded and recycled [23]. This study examined the impact of GoAstV infection on autophagy within goose kidney cells, elucidating the underlying mechanisms through both in vivo and in vitro model systems. Observations via transmission electron microscopy revealed a marked increase in autophagosomes and autolysosomes in kidney cells post-GoAstV infection, suggesting a significant activation of autophagic activity during the early stages of viral infection.

The AMPK/mTOR pathway is crucial in autophagy regulation [24]. AMPK, activated by stresses like oxidative stress and nutrient deficiency, maintains intracellular stability and inhibits mTORC1, a negative feedback regulator of autophagy [25]. This inhibition induces autophagy, which involves autophagy-related proteins (ATG proteins) that start the process by assembling autophagic vesicle membranes on organelle membranes [26]. ATG5 is crucial in both classical and non-classical autophagy, aiding autophagosome membrane formation by interacting with LC3 and PE. ATG7, an E1-like kinase, activates ATG5-ATG12 complex assembly and facilitates LC3-I and PE binding to form LC3-II [27,28,29]. Together, ATG5 and ATG7 underpin autophagy [30]. Beclin1, a central PI3K complex component, regulates autophagy by forming functional structures with various proteins [31]. AMBRA1 enhances Beclin1 activity, and its downregulation reduces autophagic activity [32]. In this study, the results indicated that GoAstV infection modulates autophagy via the AMPK/mTOR signaling pathway, evidenced by the upregulation of *AMPK* mRNA expression and the downregulation of *mTOR* mRNA expression. Moreover, the mRNA and protein levels of autophagy-related genes, including ATG5, ATG7, Beclin1, AMBRA1, LC3A, and GABARAPL, were significantly elevated in the infected group, further corroborating the enhancement in autophagy. The observed increase in the LC3B II/I ratio and the degradation of the P62 protein provided additional evidence supporting the augmentation in autophagic flux.

The temporal and dynamic differences in autophagy activation between in vivo and in vitro models are notable. In vitro, electron microscopy showed increased autophagosomes and autolysosomes at 6 hpi, peaking at 18 hpi, with viral particles in vesicles by 30 hpi. Autophagic flux was blocked up to 30 hpi, indicated by elevated LC3B II/I and P62, but normalized by 42 hpi. In contrast, the in vivo model showed a delayed response in autophagy-related protein expression. At 3 dpi, Beclin1 protein levels increased significantly, aligning with the peak viral load, while the LC3B II/I ratio rose, and P62 protein levels dropped, indicating enhanced autophagy. By 7 dpi, these indicators declined. The slower in vivo changes, compared to rapid in vitro changes, may be due to: (i) the complex stages of in vivo infection, including viral replication, spread, and immune response, which delay autophagy signaling; (ii) varied responses among different cell types that may obscure overall autophagy detection; and (iii) the need for the virus to bypass tissue barriers and immune defenses in vivo, unlike the direct infection in vitro.

3-MA functions as an autophagy inhibitor by targeting and suppressing PI3K [33]. In this study, 3-MA was employed in conjunction with GoAstV to treat primary renal tubular epithelial cells derived from geese. The objective was to examine its impact on autophagy processes induced by GoAstV infection in these cells. In the in vitro model, the co-localization of autophagosomes and viral particles in primary renal tubular epithelial cells of geese infected by GoAstV indicates that the virus might utilize autophagosomes as a replication site. Immunofluorescence assays reveal that LC3B and the viral Cap protein co-localize at 18 hpi, but this co-localization is absent at 30 hpi, potentially due to the obstruction of autophagic flux and subsequent viral clearance. Treatment with the autophagy inhibitor 3-MA significantly reduces GoAstV-induced autophagy and viral load, suggesting that autophagy may promote viral proliferation. It is noteworthy that GoAstV infection results in the disruption of autophagic flux during the logarithmic phase of viral replication, as evidenced by the accumulation of the P62 protein and an elevated LC3B II/I ratio. Following the peak of viral load, autophagic flux is restored to normal levels. This observation may be associated with the cellular defense mechanism aimed at eliminating viral particles via autophagy. The exact mechanisms by which autophagy influences GoAstV infection, especially regarding its molecular interactions with viral proliferation, remain unclear.

## 5. Conclusions

In summary, early GoAstV infection induces enhanced autophagy in goose kidney cells; autophagosomes are hijacked by the virus and used as replication factories for viral proliferation. During the logarithmic phase of viral replication, the autophagic flux in kidney cells is blocked. After viral replication peaks, the autophagic flux is restored, and the viral load rapidly decreases. The use of the autophagy inhibitor 3-MA can suppress GoAstV-induced cellular autophagy and reduce the rate of GoAstV replication. However, the mechanism underlying autophagy during GoAstV infection and its relationship to viral replication require further investigation.

## Figures and Tables

**Table 1:** The sequences of target gene primers.

Gene	Primer Sequences (5′ to 3′)
*GAPDH*	F: 5′ GGTGCTAAGCGTGTCATCATCTC 3′ R: 5′ AGACCCTCCACGATGCCAAA 3′
*BECLIN1*	F: 5′ AGCTCGACACGTCCTTCAAG 3′ R: 5′ GCCCTGACCCTCTCAAAGTC 3′
*ATG5*	F: 5′ TGCATCAAATACAGCCCTTCCT 3′ R: 5′ GCTCATGTGTTCGCTTAGCC 3′
*ATG7*	F: 5′ CACGTTTCAGCAATGCCTCC 3′ R:5′ CTCATGTCCCAGATCTCGGC 3′
*LC3A*	F: 5′ CGCGGATCGTTGTAAAGAAGT 3′ R: 5′ TCTCCTGGGAAGCATAGACCA 3′
*LC3B*	F:5′ CGAAGTCATCACCCTGAGAGAT 3′ R: 5′ TCCTCGTCCTTCTCGCTCTC 3′
*mTOR*	F: 5′ GAAGAGTGCCCGTAAGATTG 3′ R:5′ GTGGAGTTCCTGCTTGTGC 3′
*AMPK*	F: 5′ TTGTTTCCCGAAGACCC 3′ R: 5′ GAGTATGGCGAGGACGAGG 3′
*AMBRA1*	F: 5′ CCATTTCCGTGAGCCTGTCT 3′ R: 5′ CTGCTCCTGTACCTCCCTCT 3′
*GABARAPL1*	F: 5′ GACCACCCGTTCGAGTACAG 3′ R:5′ TCCAGTTGCCATAGACGCTC 3′

## Data Availability

The original contributions presented in this study are included in the article. Further inquiries can be directed to the corresponding authors.
